# Long non-coding RNA TRPM2-AS regulates microRNA miR-138-5p and PLAU (Plasminogen Activator, Urokinase) to promote the progression of gastric adenocarcinoma

**DOI:** 10.1080/21655979.2021.1995101

**Published:** 2021-12-07

**Authors:** Jun Sun, Fang Zhou, Juan Xue, Chunyan Ji, Yinzong Qu, Yuwei Pan

**Affiliations:** a Department of Gastroenterology, Hubei Provincial Hospital of Integrated Chinese and Western Medicine, Wuhan, Hubei, China; b Department of Preventive Medicine, Tianhe District Hospital of Traditional Chinese Medicine, Guangzhou, Guangdong, China

**Keywords:** TRPM2-AS, miR-138-5p, PLAU, gastric adenocarcinoma

## Abstract

Gastric adenocarcinoma (GAC) is a common malignant tumor, accounting for 95% of gastric cancers. However, the effects and regulatory mechanisms of long non-coding RNA TRPM2-AS (TRPM2-AS) in GAC have not been fully explored. Our study investigates the action mechanism of TRPM2-AS in GAC. After performing quantitative Real-Time polymerase chain reaction or western blotting, we found that the levels of TRPM2-AS and Plasminogen Activator, Urokinase (PLAU) were upregulated in GAC, whereas the level of miR-138-5p was downregulated. Cell function experiments proved that silencing TRPM2-AS suppressed proliferation and migration and induced apoptosis in GAC cells. Bioinformatic analysis and luciferase assay identified the interaction between TRPM2-AS, miR-138-5p, and PLAU. In addition, the inhibitory effect of silencing TRPM2-AS on GAC cells could be partially relieved by PLAU overexpression. In conclusion, our study revealed that TRPM2-AS sponging miR-138-5p to upregulate PLAU could contribute to GAC progression, which might be useful for identifying biomarkers for GAC therapy.

Abbreviation

Gastric adenocarcinoma (GAC); Long non-coding RNA (lncRNA); lncRNA TRPM2 antisense RNA (TRPM2-AS); Plasminogen Activator, Urokinase (PLAU); Wild-type (WT); mutant (MUT).

## Introduction

Gastric adenocarcinoma (GAC) is the most common malignant tumor originating in the stomach [1]. GAC accounting for 95% of gastric cancer invades the stomach wall and penetrates the muscular mucosa and submucosa, destroying the muscularis propria [2,3]. The combination of surgery and radiation therapy, or chemotherapy, can improve the GAC survival rate; however, its treatment and prognosis are still poor due to the chemoresistance and non-typical symptoms of early GAC [4,5]. Therefore, it is crucial to investigate the regulatory mechanisms of GAC.

Long non-coding RNAs (lncRNAs) with more than 200 nucleotides are essential in many biological processes by sponging microRNAs (miRNAs) [6–8]. In recent years, researchers have revealed that lncRNAs are closely associated with GAC, including lncRNA FOXCUT [9], lncRNA MEG3, lncRNA PMS2L2 [10]. Due to the complex mechanism of lncRNAs in GAC, identifying the mechanism associated with tumor-related lncRNAs in GAC is still challenging.

The lncRNA TRPM2 antisense RNA (TRPM2-AS) is related to the progression of multiple human cancers [11,12]. Several studies have revealed that TRPM2-AS exerts pro-tumor effects in prostate cancer [13], breast cancer [11], hepatocellular carcinoma [14]. TRPM2-AS was also shown to be a tumor promoter in gastric cancer by regulating miR-612, mitogen-activated protein kinase, and activators of transduction-3, or the miR-195/HMGA1 axis . GAC is a common gastric cancer, but the effects and regulatory mechanisms of TRPM2-AS in GAC have not been fully explored.

Together with the findings of previous study, we suspected that TRPM2-AS might be a key factor to participate in the progression of GAC. Therefore, this study aims to elucidate the function of TRPM2-AS in GAC using bioinformatic analysis and cell function experiments. Our findings provide new insights into the potential therapeutic and diagnostic targets of GAC.

## Materials and methods

### Clinical samples collection and cell culture

GAC samples and paired adjacent normal tissues were collected from 32 patients, aged 41–72 years old (56 ± 15 years old), diagnosed with GAC in our hospital between April 2018 and May 2020. The inclusion criteria for study selection were as follows [1]: patients diagnosed with GAC by pathology or histology [2]; patients subjected to surgery. The exclusion criteria for study selection were as follows [1]: prior history of gastric cancer treatment [2]; other diagnosed clinical disorders. After resection, the GAC and paired adjacent normal tissues were immediately frozen in liquid nitrogen. Our study was approved by the Ethics Committee of Hubei Provincial Hospital of Integrated Chinese and Western Medicine (Approved No. of ethic committee: 2,018,317), and written informed consent was obtained before sample collection. Clinical information of the 32 patients is listed in Supplementary Table I.

Two GAC cell lines, AGS (BNCC338141) and MKN-7 (BNCC342521), and the human gastric mucosa cell line GES-1 (BNCC337970), were purchased from BeNa Culture Collection (BNCC, China). GES-1 and MKN-7 cell lines were cultured in Roswell Park Memorial Institute-1640 (RPMI-1640) medium (Gibco, USA), and AGS cells were cultured in 90% Ham’s F-12 K (F-12 K) medium (Gibco, USA). All cells were cultured with 10% fetal bovine serum (FBS, Gibco, USA) at 5% CO_2_ and 37°C.

### Quantitative real-time polymerase chain reaction (qRT-PCR)

Total RNA was extracted using TRIzol reagent (Invitrogen, USA) and quantified using a NanoDrop 2000 (Thermo Fisher Scientific, USA). Then, the ReverTra Ace qPCR RT Kit (Toyobo, Japan) was used to reverse transcribe 1 μg RNA into cDNA according to the manufacturer’s instructions. qRT-PCR was performed on ABI7500 instrument (Applied Biosystems, China) using the SYBR-Green Master Mix (Life Technologies, USA) with 10 μL reaction volume and following cycling condition: 25°C for 10 min, 37°C for 30 min, and 95°C for 5 min. The results of qRT-PCR were confirmed by agarose gel electrophoresis. The 2^–∆∆Ct^ method [15] was applied to calculate the relative expression of the internal control GAPDH for lncRNA and mRNA, and U6 for miRNA. The primer sequences used in our study are listed in Supplementary Table II.

### Fluorescence in situ hybridization (FISH) assay

A Cy3-labeled TRPM2-AS complementary DNA probe mix was purchased from Ribo Ltd. (China) to perform FISH assay according to previous study [16]. Briefly, 20 thousand GAC cells were plated into a 15 mm dish for 24 h of incubation. Then, the cells were fixed 4% paraformaldehyde, permeabilized in acetone and methanol solution (1:1), and blocked with 200 μL pre-hybridization buffer. Next, 20 μM probe mix was added into the dish for incubation overnight. After washing the dish using washing buffer, the nuclei were stained using DAPI. Images were photographed using a confocal microscope (Olympus, Japan) at 400x magnification.

### Cell transfection

The two short interfering RNAs (siRNAs) targeting TRPM2-AS (si-TRPM2-AS-1 and si-TRPM2-AS-2), negative control siRNA (si-NC), miR-138-5p mimic, miR-138-5p inhibitor, and negative control of mimic (mimic-NC) or inhibitor (inhibitor-NC) were obtained from GenePharma (China). Plasminogen Activator, Urokinase (PLAU) overexpression vectors (PLAU-OE) were constructed by OBiO Technology (China). The full-length PLAU was amplified and inserted into the Hind III/Xho I sites of pcDNA 3.1 with the CMV promoter, and the stable cells were screened by neomycin resistance. The empty vector pcDNA 3.1 was used as the negative control of PLAU-OE. The 50 nM si-TRPM2-AS-1, 50 nM si-TRPM2-AS-2, 50 nM PLAU-OE, 50 nM pcDNA 3.1, 50 nM miR-138-5p mimic, and 50 nM miR-138-5p inhibitor were transfected into AGS and MKN-7 cells using Lipofectamine 2000 (Invitrogen, USA) according to the manufacturer’s instructions. After transfection for 48 h, qRT-PCR was performed to assess transfection efficiency.

### The detection of cell proliferation

Cell viability was detected using the Cell Counting Kit-8 (CCK-8) solution (Dojindo, Japan) to assess cell proliferation according to previous study [17]. Following transfection, 2000 cells were briefly seeded into 96-well plates with 100 μL of medium. At the indicated timepoint (0-day, 1-day, 2-day, and 3-day), 10 μL/well CCK-8 solution was added to the plates and incubated for 2 h at 37°C. Finally, the absorbance was measured at 450 nm using a microplate reader (Detie Lab, China).

### The detection of cell apoptosis

Flow cytometry was used to detect cell apoptosis using the FITC Annexin V Apoptosis Detection Kit (BD, USA) according to the manufacturer’s protocol. Six thousand transfected cells were collected in 100 µL binding buffer, and then incubated with 5 µL Annexin V-FITC and 5 µL PI for 20 min in the dark. The cell apoptosis rate was detected by flow cytometry (Beckman, USA) and analyzed using FlowJo software (Tree Star, USA).

### The detection of cell migration

A transwell migration assay was performed to identify the migration of GAC cells according to previous study [18]. After transfection, 2000 cells were seeded into the upper transwell chamber with serum-free media, whereas the bottom chamber was added to the medium containing 10% FBS. After 24 h of incubation, the cells that failed to penetrate the membranes were removed, and the migratory cells were fixed at 22°C using 4% paraformaldehyde for 30 min. Then, the migratory cells were stained with 0.5% crystal violet at 22°C for another 30 min. Images of migratory cells were photographed using a light microscope at 250x magnification.

### The detection of protein expression

Western blotting was used to detect the expression of the PLAU protein according to previous study [19]. Total protein from transfected cells was isolated using 200 µL of RIPA lysis buffer (Beyotime, China). Then, 20 µg of protein was added to 12% sodium dodecyl sulfate-polyacrylamide gel electrophoresis (SDS-PAGE) gels and transferred to polyvinylidene fluoride (PVDF) membranes. After blocking the membranes with 5% nonfat milk in TBST, the membranes were incubated with PLAU antibody (Cat#: ab169754, Abcam, USA) or GAPDH antibody (Cat#: ab9485, Abcam, USA) at 4°C for 12 h. Next, the HRP anti-rabbit IgG antibody (Cat#: ab270144, Abcam, USA) was incubated at 22°C for 3 h. After washing the membranes, SuperEnhanced chemiluminescence detection reagent (Applygen, China) was added to the membranes for 15 min incubation, and the damp blot was covered with plastic wrap. The blot was exposed to an X-ray film.

### Bioinformatics analysis

Two mRNA expression profiles (GSE79973 and GSE103236) were obtained from GEO DataSets (https://www.ncbi.nlm.nih.gov/gds/?term=) to screen the upregulated genes in GAC samples with adj.P < 0.05, and logFC>1. WebGestalt (http://www.webgestalt.org/option.php) was used to perform gene ontology (GO) enrichment of the upregulated genes. The expression of screened genes in the GAC samples was identified using The Cancer Genome Atlas (TCGA) database. StarBase analyzed the correlation between key genes and TRPM2-AS. Finally, starBase and TargetScan were utilized to predict the miRNA binding to TRPM2-AS and key genes, respectively.

### The detection of targeting relationship

A luciferase assay was used to confirm the relationship between TRPM2-AS, miR-138-5p, and PLAU according to the previous study [16]. The wild-type (WT) TRPM2-AS and WT PLAU 3ʹUTR containing the binding sites of miR-138-5p were constructed to psiCHECK2 vectors by Shanghai Tuoran (China). The mutant (MUT) TRPM2-AS and MUT PLAU 3ʹUTR without the binding sites of miR-138-5p were also constructed to psiCHECK2 vectors by Shanghai Tuoran (China). After AGS and MKN-7 cells co-transfected with mimic-NC/mimic and WT/MUT TRPM2-AS or PLAU 3ʹUTR for 48 hours, the activities of firefly and Renilla were measured by the Dual-Luciferase Reporter Assay System (Promega, USA). Firefly luciferase activity was used to normalize relative luciferase activity.

### Statistical analysis

The data (mean ± SD) from at least three independent experiments were analyzed using GraphPad Prism 8.0 (GraphPad Prism, USA). The paired student’s t-test with Kolmogorov-Smirnov test to assess the normality distribution of the data, and ANOVA with Dunnett’s or Tukey’s post hoc tests were performed to compare the statistical difference between two and multiple groups, respectively. Statistical significance was set at P < 0.05.

## Results

In our study, we hypothesized that TRPM2-AS might be participated in GAC progression by regulating miR-138-5p/PLAU axis. To explore the roles of TRPM2-AS, miR-138-5p, and PLAU in GAC cells, we performed a series of cell function experiments. Our data suggested that TRPM2-AS was upregulated in GAC, and silencing TRPM2-AS inhibits the GAC progression in vitro by sponging miR-138-5p to upregulate PLAU. In summary, our findings proved the function of TRPM2-AS/miR-138-5p/PLAU axis in GAC cells, which might provide novel targets for GAC therapy.

### The effect of TRPM2-AS on GAC

**Figure 1.**
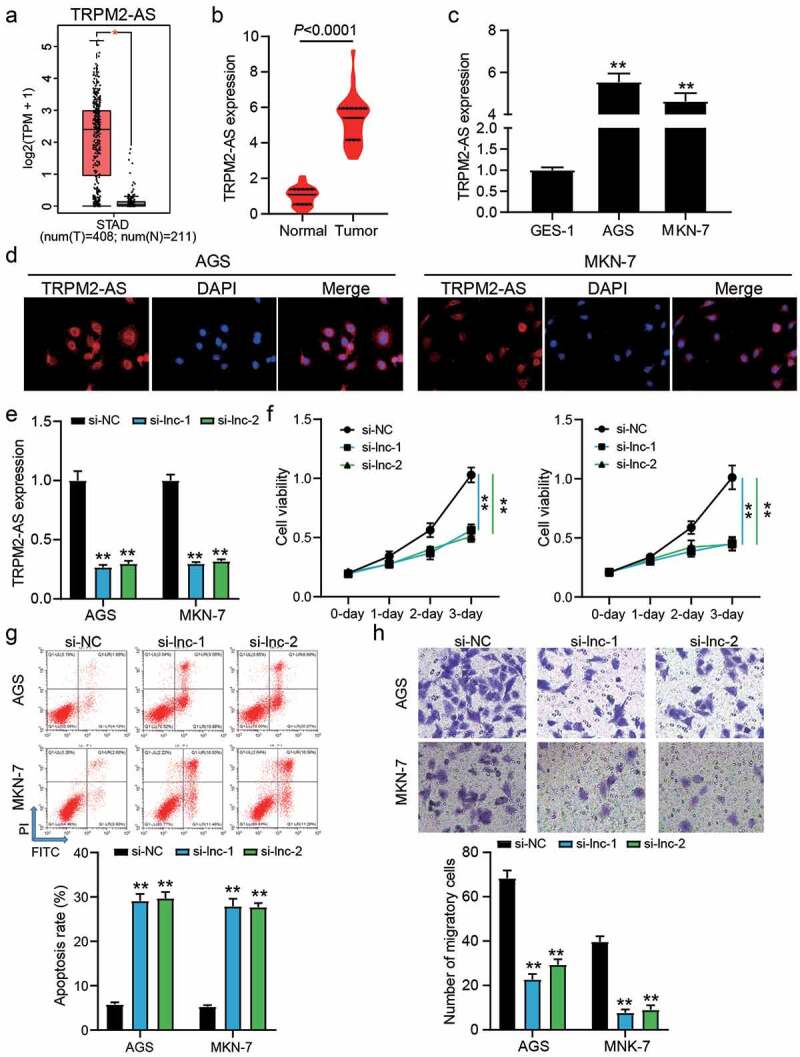
The effect of TRPM2-AS on GAC cells. (a) The expression of TRPM2-AS in normal and STAD samples by GEPIA analysis. STAD, stomach adenocarcinoma. *P < 0.01. (b) The expression of TRPM2-AS in tumor and adjacent normal samples from GAC patients. N = 32, paired student’s t-test. (c) The expression of TRPM2-AS in human gastric mucosa cell line GES-1 and GAC cell lines (AGS and MKN-7). N = 3, **P < 0.001 VS. GSE-1 using ANOVA with Dunnett’s post hoc test. (d) localization of TRPM2-AS was identified using FISH assay. (e) The transfection efficiency of siRNAs targeting TRPM2-AS in AGS and MKN-7 cells was detected by qRT-PCR. (f) The cell proliferation in transfected AGS and MKN-7 cells was detected by CCK8 assay. (g) The apoptosis rate in transfected AGS and MKN-7 cells was detected by flow cytometry. (h) The cell migration in transfected AGS and MKN-7 cells was detected by transwell assay. (e-h) si-lnc-1 and si-lnc-2 were two siRNAs of TRPM2-AS. si-NC, negative control of siRNAs of TRPM2-AS. N = 3, **P < 0.001 VS. si-NC using ANOVA with Tukey’s post hoc test

According to the data from GEPIA, it was found that TRPM2-AS was upregulated in 408 GAC samples compared with 211 normal samples (Figure 1(a)). Our qRT-PCR results also demonstrated the high expression of TRPM2-AS in 32 GAC samples compared with adjacent normal samples (*P* < 0.0001, Figure 1(b)), as well as TRPM2-AS was upregulated by around 5-fold in GAC cells (AGS and MKN-1) compared with human gastric mucosa cell GES-1 (*P* < 0.001, Figure 1(c)). The FISH assay proved that TRPM2-AS mainly located in the cytoplasm of GAC cells (Figure 1(d)), suggesting that TRPM2-AS might participate in the GAC development by regulating the expression of targets at the post transcriptional level. Then, two siRNAs targeting TRPM2-AS (si-lnc-1 and si-lnc-2) were transfected into two GAC cells (AGS and MKN-7), in which TRPM2-AS expression was reduced by 70% in si-lnc-1 and si-lnc-2 groups (*P* < 0.001, Figure 1(e)). The CCK-8 assay showed that silencing TRPM2-AS inhibited cell proliferation (*P* < 0.001, figure 1(f)), but the flow cytometry assay showed that silencing TRPM2-AS enhanced the apoptosis rate by 5-fold in AGS and MKN-7 cells (*P* < 0.001, Figure 1(g)). For cell migration, the number of migratory cells was reduced by approximately 70% when AGS and MKN-7 cells were transfected with si-lnc-1 or si-lnc-2 (*P* < 0.001, Figure 1(h)). These data suggest that silencing TRPM2-AS could inhibit the malignancy of GAC cells.

### miR-138-5p and PLAU were predicted as the downstream of TRPM2-AS

**Figure 2.**
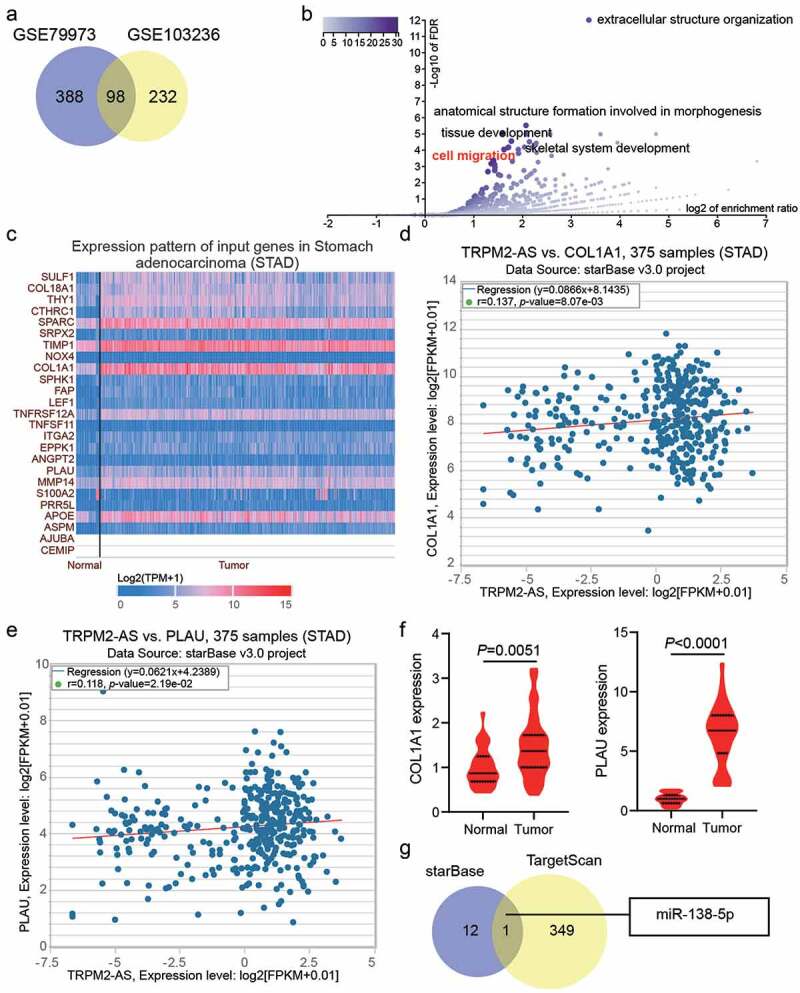
Bioinformatics analysis predicted that miR-138-5p and PLAU were the downstream of TRPM2-AS. (a) 98 upregulated genes were screened from GSE79973 and GSE103236 with adj.P < 0.05 and logFC>1. (b) cell migration involving 25 genes was identified as the key biological process by WebGestalt analysis. (c) The expression pattern of 25 genes in stomach adenocarcinoma according to TCGA data. (d) The correlation between TRPM2-AS and COL1A1 in stomach adenocarcinoma according to starBase analysis. STAD, stomach adenocarcinoma. (e) The correlation between TRPM2-AS and PLAU in stomach adenocarcinoma according to starBase analysis. STAD, stomach adenocarcinoma. (f) The expression of COL1A1 and PLAU in tumor and adjacent normal samples from GAC patients. N = 37, paired student’s t-test. (g) miR-138-5p was predicted to bind to TRPM2-AS and PLAU by starBase for TRPM2-AS and TargetScan for PLAU

GSE79973 and GSE103236 were mRNA expression profiles to screen the upregulated genes in GAC samples with adj.P < 0.05, and logFC>1, finding that 93 upregulated common genes overlapped from GSE79973 and GSE103236 (Figure 2(a)). The 93 upregulated genes were uploaded to WebGestalt for GO enrichment, and 25 genes were found to be involved in cell migration (Figure 2(b)). According to TCGA database, 6 of the 25 genes (SULF1, CTHRC1, SPARC, TIMP1, COL1A1, and PLAU) were overexpressed in the GAC samples (Figure 2(c)). However, the expression of only two of the six genes (COL1A1 and PLAU) was positively correlated with TRPM2-AS in GAC samples based on starBase analysis (Figures 2(d,e)). In our clinical samples, PLAU exhibited higher expression levels in GAC samples compared with COL1A1 (6.3-fold VS. 1.5-fold); therefore, PLAU was selected as the key gene for exploration (figure 2(f)). Using starBase to predict miRNAs for TRPM2-AS and TargetScan to predict miRNAs for PLAU, only one miRNA, miR-138-5p, could bind to TRPM2-AS and PLAU (Figure 2(g)). Bioinformatic analysis predicted that miR-138-5p and PLAU might be downstream of TRPM2-AS.

### The targeting relationship between TRPM2-AS, miR-138-5p and PLAU in GAC cells

**Figure 3.**
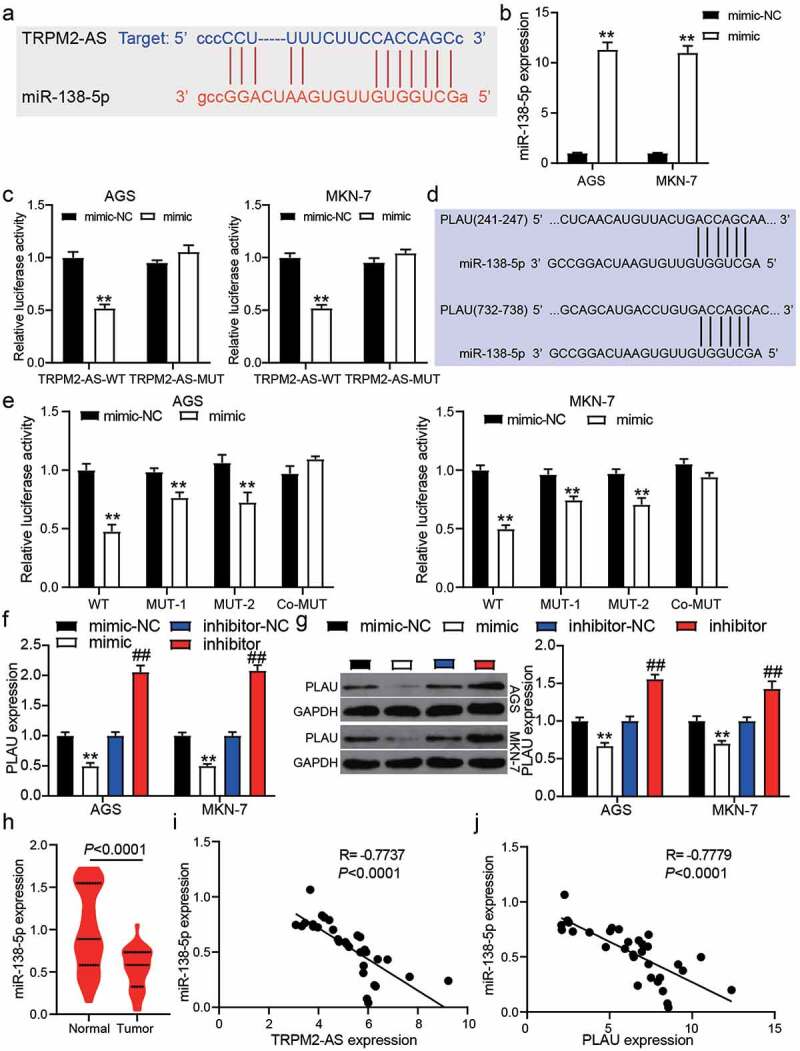
miR-138-5p and PLAU were the downstream of TRPM2-AS. (a) The binding site between TRPM2-AS and miR-138-5p was predict by starBase. (b) The transfection efficiency of miR-138-5p mimic in AGS and MKN-7 cells was detected by qRT-PCR. NC, negative control. mimic, miR-138-5p mimic. N = 3, **P < 0.001 VS. mimic-NC using ANOVA with Tukey’s post hoc test. (c) The targeting relationship between TRPM2-AS and miR-138-5p was identified by luciferase assay. WT, wild-type. MUT, mutant. NC, negative control. mimic, miR-138-5p mimic. N = 3, **P < 0.001 VS. mimic-NC using ANOVA with Tukey’s post hoc test. (d) The two binding sites between PLAU and miR-138-5p were predict by TargetScan. (e) The targeting relationship between PLAU and miR-138-5p was identified by luciferase assay. WT, wild-type. MUT-1 and MUT-2 were the two mutant sites for the two binding sites in PLAU 3ʹUTR. NC, negative control. mimic, miR-138-5p mimic. N = 3, **P < 0.001 VS. mimic-NC using ANOVA with Tukey’s post hoc test. (f-g) The expression of PLAU in transfected AGS and MKN-7 cells was detected by qRT-PCR (f) and western blotting (g). N = 3, **P < 0.001 VS. mimic-NC and ##P < 0.001 VS. inhibitor-NC using ANOVA with Tukey’s post hoc test. (h) The expression of miR-138-5p in tumor and adjacent normal samples from GAC patients. N = 37, paired student’s t-test. (i) The correlation between TRPM2-AS and miR-138-5p in tumor samples from GAC patients. (j) The correlation between PLAU and miR-138-5p in tumor samples from GAC patients

The binding sites between TRPM2-AS and miR-138-5p were predicted using starBase (Figure 3(a)). To identify their targeting relationship, we first transfected the miR-138-5p mimic into AGS and MKN-7 cells, finding that miR-138-5p mimic significantly upregulated the miR-138-5p expression (*P* < 0.001, Figure 3(b)). The luciferase assay showed that the luciferase activity was suppressed only by the co-transfection of TRPM2-AS-WT and miR-138-5p mimic (*P* < 0.001, Figure 3(c)), suggesting a targeting relationship between TRPM2-AS and miR-138-5p. As shown in Figure 3(d), TargetScan predicted two binding sites between the PLAU 3ʹUTR and miR-138-5p. After performing the luciferase assay, we found that co-transfection of PLAU-WT and miR-138-5p mimics suppressed 50% luciferase activity in GAC cells, and the luciferase activity could be slightly suppressed by co-transfection of PLAU-MUT1/MUT2 and miR-138-5p (*P* < 0.001, Figure 3(e)). qRT-PCR and western blot assays further confirmed that PLAU expression could be suppressed by miR-138-5p mimic and promoted by miR-138-5p inhibitor (*P* < 0.001, Figures 3(f) and 3 G). In our clinical samples, miR-138-5p expression was reduced by 40% in 32 GAC samples compared with adjacent normal samples (*P* < 0.0001, Figure 3(h)), and its expression was negatively correlated with TRPM2-AS expression and PLAU expression in GAC samples by Pearson’s correlation analysis (Figures 3(i,j)). Our findings indicate that TRPM2-AS sponges miR-138-5p, which could target PLAU in GAC cells.

### The inhibitory effect of silence TRPM2-AS was partly relieved by miR-138-5p inhibitor

**Figure 4.**
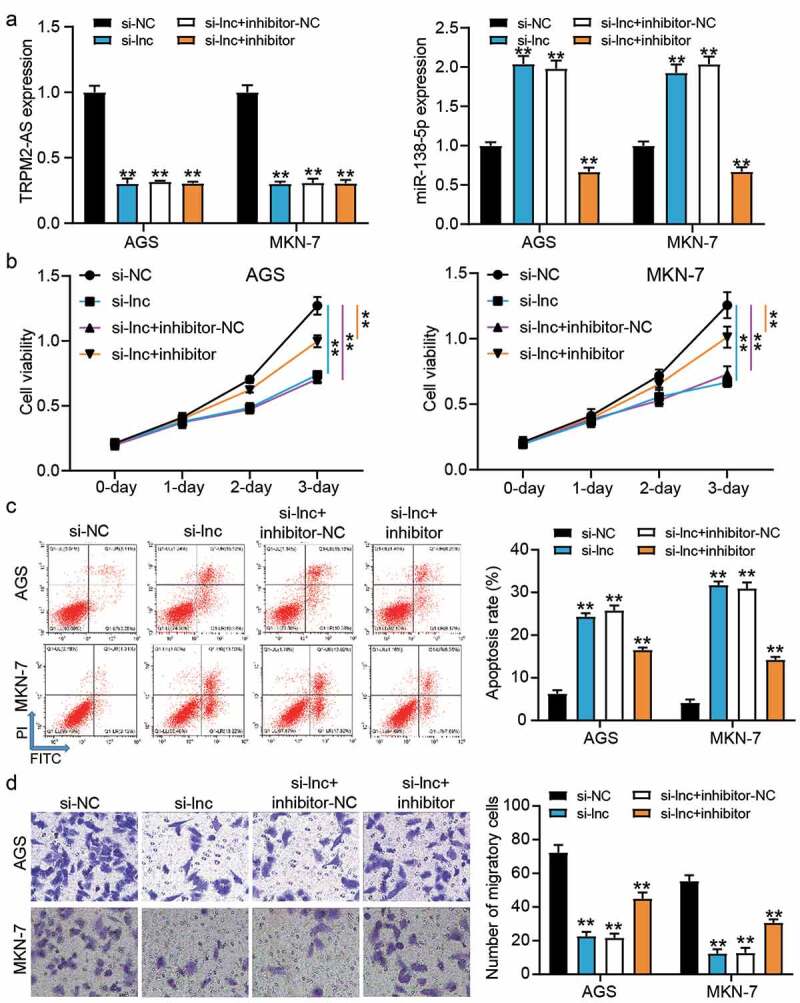
miR-138-5p inhibitor partly relived the inhibitory effect of silence TRPM2-AS on GAC cells. (a) The expression of TRPM2-AS and miR-138-5p in transfected AGS and MKN-7 cells was detected by qRT-PCR. (b) The cell viability in transfected AGS and MKN-7 cells was detected by CCK8 assay. (c) The apoptosis rate in transfected AGS and MKN-7 cells was detected by flow cytometry. (d) The cell migration in transfected AGS and MKN-7 cells was detected by transwell assay. si-NC, negative control of siRNA of TRPM2-AS. si-lnc, siRNA of TRPM2-AS. inhibitor-NC, negative control of miR-138-5p inhibitor. inhibitor, miR-138-5p inhibitor. N = 3, **P < 0.001 VS. si-NC using ANOVA with Tukey’s post hoc test

Because of the targeting relationship between TRPM2-AS and miR-138-5p, we performed a series of cell function experiments to confirm the effect of their relationship on GAC cells. After transfecting si-TRPM2-AS and miR-138-5p inhibitor into GAC cells, it was found that silencing TRPM2-AS upregulated miR-138-5p expression by almost 2-fold but this effect could be overturned by co-transfection of si-TRPM2-AS and miR-138-5p inhibitor (*P* < 0.001, Figure 4(a)). However, miR-138-5p inhibitor could not affect TRPM2-AS expression in GAC cells, suggesting that TRPM2-AS was upstream of miR-138-5p. The CCK-8 assay proved that the decrease in cell proliferation caused by si-TRPM2-AS could be partly relieved by co-transfection with miR-138-5p inhibitor (*P* < 0.001, Figure 4(b)). The results from flow cytometry showed that the increase of cell apoptosis induced by si-TRPM2-AS could be reduced by co-transfection with miR-138-5p inhibitor (*P* < 0.001, Figure 4(c)). Moreover, the inhibitory effect of silencing TRPM2-AS on cell migration could be partly recovered by co-transfection with the miR-138-5p inhibitor (*P* < 0.001, Figure 4(d)). These data suggested that the miR-138-5p inhibitor partly recovered the inhibitory effect of silencing TRPM2-AS on GAC cells.

### The inhibitory effect of silence TRPM2-AS was partly relieved by PLAU overexpression

**Figure 5.**
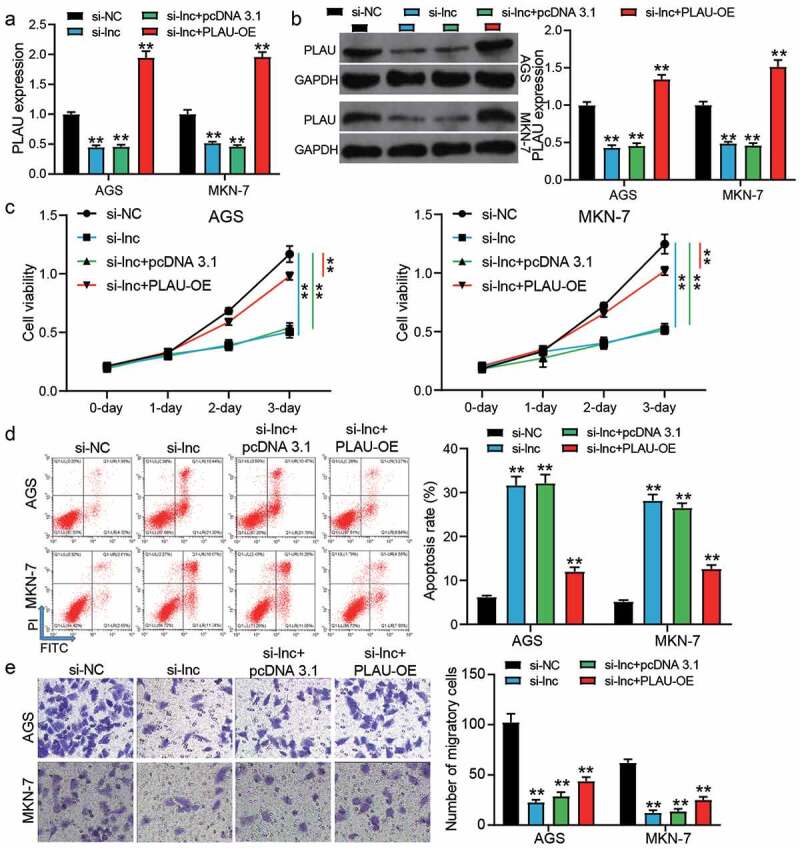
PLAU overexpression partly relived the inhibitory effect of silence TRPM2-AS on GAC cells. (a) The expression of PLAU in transfected AGS and MKN-7 cells was detected by qRT-PCR. (b) The expression of PLAU in transfected AGS and MKN-7 was detected by western blotting. (c) The cell proliferation in transfected AGS and MKN-7 cells was detected by CCK8 assay. (d) The apoptosis rate in transfected AGS and MKN-7 cells was detected by flow cytometry. (e) The cell migration in transfected AGS and MKN-7 cells was detected by transwell assay. si-NC, negative control of siRNA of TRPM2-AS. si-lnc, siRNA of TRPM2-AS. pcDNA 3.1, negative control of PLAU-OE. PLAU-OE, PLAU overexpression vectors. N = 3, **P < 0.001 VS. si-NC using ANOVA with Tukey’s post hoc test

si-TRPM2-AS and PLAU overexpression vectors (PLAU-OE) were transfected into AGS and MKN-7 cells to verify the effect of the interaction between TRPM2-AS and PLAU on GAC cells. The results of qRT-PCR and western blotting showed that si-TRPM2-AS reduced PLAU expression by ~50% in AGS and MKN-7 cells, but co-transfection of si-TRPM2-AS and PLAU-OE enhanced PLAU expression (*P* < 0.001, Figures 5(a,b)). The CCK-8 assay showed that co-transfection of si-TRPM2-AS and PLAU-OE partly relieved the inhibitory effect of silencing TRPM2-AS on cell proliferation (*P* < 0.001, Figure 5(c)). Cell apoptosis detected by flow cytometry suggested that the elevated apoptosis rate in the si-TRPM2-AS group was reduced by co-transfection with PLAU-OE (*P* < 0.001, Figure 5(d)). As for cell migration, the transwell assay revealed that the depressed cell migration caused by silencing TRPM2-AS could be elevated by co-transfection with PLAU-OE (*P* < 0.001, Figure 5(e)). These data proved that PLAU overexpression could partly relieve the negative effect of silencing TRPM2-AS on GAC cells.

## Discussion

An increasing number of studies have reported the significant effect of lncRNAs on GAC tumorigenesis, indicating that lncRNAs may be useful therapeutic targets for GAC [9,20]. TRPM2-AS has been shown to contribute to gastric cancer progression and radioresistance in vivo and in vitro [16,21,22]. However, the mechanism of action of TRPM2-AS on GAC remains unclear. We explored the effect of TRPM2-AS on GAC. In vitro experiments revealed that TRPM2-AS contributed to cell proliferation and cell migration but inhibited cell apoptosis, which is consistent with previous studies on TRPM2-AS in gastric cancer. In addition, the positive effect of TRPM2-AS on GAC cells could be regulated by the miR-138-5p/PLAU axis due to their targeting relationship.

The function of lncRNAs has been reported to act as endogenous miRNA sponges to regulate the target genes of miRNAs, thereby participating in multiple biological processes [23]. It was reported that TRPM2-AS located in the cytoplasm could sponge miR-195 to promote the expression of HMGA1 (target gene of miR-195), thereby playing a tumor promoter role in gastric cancer [22]. We found that TRPM2-AS contributed to GAC progression by sponging miR-138-5p to upregulate PLAU, which was different from the previous study. According to the previous studies, miR-138-5p suppressed the proliferation and migration of gastric cancer cells [24,25], which had the contrary effect of TRPM2-AS in gastric cancer. Here, we further confirmed the inhibitory effect of miR-138-5p on GAC by proving that it could be sponged by TRPM2-AS and target PLAU in GAC cells. Our findings enrich the mechanism by which miR-138-5p regulates the progression of GAC.

PLAU, another name Urokinase-type plasminogen activator, proteolyzes proteins associated with ECM remodeling and activates growth factors [26]. Previous studies revealed that PLAU is an oncogene in multiple cancers, including colorectal cancer [27], pancreatic cancer [19], breast cancer [28]. In gastric cancer, Zhang et al. [29] found that a poor prognosis was linked to PLAU overexpression; however, they did not explore the function of PLAU in gastric cancer. In our study, we investigated the effect of PLAU on GAC cells. Results indicated PLAU overexpression relieved the inhibitory effect of TRPM2-AS on GAC cells, suggesting that PLAU promotes the malignancy of GAC cells.

Our study revealed that TRPM2-AS plays a positive role in GAC cells by sponging miR-138-5p to upregulate PLAU. However, the effect of the TRPM2-AS/miR-138-5p/PLAU axis on GAC in vivo has not been explored, and needs further investigation. Meanwhile, the regulation of TRPM2 is complex in GAC, and other regulatory factors need to be deeply explored by using multiple bioinformatic analyses such as WCGNA. Moreover, the limited number of clinical samples in our study is not enough to support the significance of the TRPM2-AS/miR-138-5p/PLAU axis in GAC, or confirm its usefulness in prognosis. In future, we will continue to solve these key problems in depth.

## Conclusion

Our study identified a novel mechanism by which TRPM2-AS contributes to GAC proliferation and invasion by regulating miR-138-5p/PLAU in vitro. Our findings provide new insights into the therapeutic and diagnostic targets of GAC.

## Supplementary Material

Supplemental Material

## Data Availability

The datasets used and/or analyzed during the current study are available from the corresponding author on reasonable request.

## Highlights

1. TRPM2-AS promotes the progression of gastric adenocarcinoma in vitro.
2. TRPM2-AS could sponge miR-138-5p to enhance PLAU expression in gastric adenocarcinoma cells.
3. TRPM2-AS promotes the progression of gastric adenocarcinoma by miR-138-5p/PLAU axis.
