# Supplementary material for: Long non-coding RNA TRPM2-AS regulates microRNA miR-138-5p and PLAU (Plasminogen Activator, Urokinase) to promote the progression of gastric adenocarcinoma
Source: Bioengineered. 2021 Dec 7;12(2):9753–65. doi: 10.1080/21655979.2021.1995101 (PMC8809918; doi:10.1080/21655979.2021.1995101)
Supplement: Supplemental Material [file KBIE_A_1995101_SM1357.zip › Supplementary table II_revised.docx]

**Supplementary table II** The sequences of the primers used in this study

| **Primer** | **Sequences** |
| --- | --- |
| **TRPM2-AS** | Forward: 5'-CGTGACCAGGTTCAGACACA-3' |
|  | Reverse: 5'-TGGGCAGTTTGGTTCTGGTT-3' |
| **COL1A1** | Forward: 5'-ACGTCCTGGTGAAGTTGGTC-3' |
|  | Reverse: 5'-ACCAGGGAAGCCTCTCTCTC-3' |
| **PLAU** | Forward: 5'-TGTGCATGGGTGAAGGGAGAGC-3' |
|  | Reverse: 5'-GTGGCAGTCTGTGGGTCTC-3' |
| **miR-138-5p** | Forward: 5'-AGCTGGTGTTGTGAATCAGGCCG-3'  Reverse: 5'-TGGTGTCGTGGAGTCG-3' |
| **GAPDH** | Forward: 5'-CGAGCCACATCGCTCAGACA -3' |
|  | Reverse: 5'-GTGGTGAAGACGCCAGTGGA -3' |
| **U6** | Forward: 5'-CTCGCTTCGGCACA-3' |
|  | Reverse: 5'-AACGCTTCACGAATTTGCGT-3' |
