# Supplementary material for: Long non-coding RNA TRPM2-AS regulates microRNA miR-138-5p and PLAU (Plasminogen Activator, Urokinase) to promote the progression of gastric adenocarcinoma
Source: Bioengineered. 2021 Dec 7;12(2):9753–65. doi: 10.1080/21655979.2021.1995101 (PMC8809918; doi:10.1080/21655979.2021.1995101)
Supplement: Supplemental Material [file KBIE_A_1995101_SM1357.zip › Supplementary table I_revised.docx]

**Supplementary table I** The baseline characteristics of GAC patients.

| **Characteristics** | **N=32** |
| --- | --- |
|  |  |
| **Age (years)** |  |
| ≤56 | 17 |
| >56 | 15 |
| **Sex** |  |
| Male | 16 |
| Female | 16 |
| **TNM stage** |  |
| I | 7 |
| II | 8 |
| III | 12 |
| IV | 5 |
| **LN metastasis** |  |
| Positive | 18 |
| Negative | 14 |
